# A Hitchhiker Story? Exploring HDL as an Overlooked Vitamin D Carrier

**DOI:** 10.1016/j.cdnut.2025.107499

**Published:** 2025-06-30

**Authors:** Jennifer D Bean, Catherine A Peterson

**Affiliations:** Division of Food, Nutrition, and Exercise Science, University of Missouri-Columbia, Columbia, MO, United States

**Keywords:** Vitamin D, cholecalciferol, lipoprotein, high-density lipoproteins, absorption, trafficking

## Abstract

**Background:**

Twenty-five-hydroxyvitamin D, used to assess vitamin status, is the primary circulating form of vitamin D having a half-life measured in weeks, and 1,25-dihydroxyvitamin D is the active metabolite having a half-life measured in hours. Although there has been a preponderance of research on the roles of the active metabolite in health, there remains a limited understanding of the transport of the true vitamin forms, ergocalciferol and cholecalciferol.

**Objective:**

This study proposes an alternative mechanism for vitamin D transport, hypothesizing that high-density lipoprotein (HDL) facilitates its movement from the intestine to the liver using cholesterol transporters.

**Methods:**

This perspective challenges the sole reliance on the classical chylomicron pathway, proposing an alternative mechanism based on vitamin D's striking structural similarity to cholesterol. Both are steroids sharing key features, suggesting analogous intestinal absorption and transport routes. Evidence indicates vitamin D utilizes known enteric cholesterol transporters for uptake. We hypothesize that high-density lipoprotein (HDL) functions as an important interorgan carrier, facilitating vitamin D's movement from the intestine to the liver, using shared cholesterol transporters. This proposed HDL-mediated delivery is particularly relevant given vitamin D-binding protein's lower affinity for vitamin D compared with 25-hydroxyvitamin D.

**Results:**

This mechanism offers a plausible explanation for observed correlations between vitamin D status (plasma 25-hydroxyvitamin D concentrations) and factors affecting HDL cholesterol concentration (HDLc). Modulators that increase HDLc (e.g., fibrates, oral contraceptives, and exercise) are associated with improved vitamin D status, whereas those that decrease HDLc or inhibit vitamin D absorption (e.g., smoking and ezetimibe) are linked to lower vitamin D status. Although current human data are largely correlative and confounded by numerous factors affecting vitamin D status, the consistent associations warrant further investigation into vitamin D’s precise trafficking among lipoprotein fractions.

**Conclusions:**

Re-evaluating vitamin D’s absorption and transport to include a role for HDL holds implications for optimizing vitamin D status.

## Introduction

The relationship between vitamin D status (VitD status), defined as the concentration of the vitamin D metabolite 25-hydroxyvitamin D (25OHD) in the serum, and various health outcomes has garnered significant attention in recent years [1]. Although the metabolites of vitamin D (VITD), especially 25OHD and 1,25-dihydroxyvitamin D, are essential for numerous physiological processes, including bone health and immune function, the indispensable foundational vitamins are cholecalciferol (D3) or ergocalciferol (D2). Humans obtain D3 and D2 from diet or obtain D3 from the transformation of 7-dehydrocholesterol by UV light (UVB) [2]. For clarity, it is important to note that here the term “VITD” will refer to both cholecalciferol (D3) and ergocalciferol (D2), whereas discussions of “VitD status” reflect total 25OHD serum concentration, which includes both D3 and D2 metabolite forms. Characterizing VITD’s movement from the intestine to the liver, the primary site of 25OHD production, is important for fully understanding its biological functions.

The initial handling of VITD in the gut warrants specific attention due to its striking structural resemblance to cholesterol. Unlike other fat-soluble vitamins, VITD shares the 4-ring gonane skeleton with cholesterol, and both possess a hydroxyl group at the third carbon position, highlighting their profound biochemical kinship within the broader steroid family (despite VITD's characteristic secosteroid structure typified by the B-ring cleavage). This chemical similarity suggests VITD's intestinal processing may parallel that of free cholesterol, which is *1*) effluxed, *2*) packaged into chylomicrons (either on the surface or esterified in the core), or *3*) directly incorporated into HDL [3].

The prevailing understanding of VITD absorption and subsequent chylomicron-mediated transport to the liver persists, yet the liver's integral role in both cholesterol and HDL metabolism presents an intriguing alternative or complementary transport pathway for VITD. This is particularly relevant because of its lower binding affinity (compared with its hydroxylated metabolites) for vitamin D-binding protein (DBP), the dominant carrier protein which is also made in the liver [4]. Common transport mechanisms are likely employed in both the intestine and liver for VITD and cholesterol, given their shared structural features. Intestinal absorption of VITD from micelles, such as cholesterol, involves known cholesterol transporters [5]. HDL may then function as an important interorgan carrier of VITD to the liver, where VITD metabolism begins, potentially using the same cholesterol transporters. This proposed mechanism may explain the observed correlations between VitD status and factors affecting HDL cholesterol concentration (HDLc).

## Challenges to the Classical VITD Intestinal Absorption Model

Classically, VITD is presumed to be trafficked through the enterocyte and packaged into triglyceride-rich lipoproteins (TRLs) such as sterol esters, even though robust evidence for this is limited. In fact, early work by Blomstrand and follow-up studies by Blomhoff clearly demonstrate radiolabeled D3 is not esterified within the enterocyte [6,7]. Furthermore, the most comprehensive study of intestinal absorption, now 30 y old, suggests VITD might follow a different path than cholesterol esters once inside the enterocyte [8]. Reboul et al. [5] were the first to show that D3 indeed uses known enteric cholesterol transporters for both uptake and efflux. Although some research using large pharmacologic doses of radiolabeled D3 describes a passive absorption pathway leading to lymphatic release, presumably within chylomicrons, important details remain unresolved [6,9,10] leaving key questions regarding VITD’s specific integration and transport within lipoproteins.

These evidentiary gaps and the compelling structural similarities led Reboul to propose an additional absorption pathway for dietary D3. Building on her pioneering findings showing D3 is absorbed via known cholesterol transporters, Scavenger Receptor Class B Type 1 (SR-B1) and Niemann-Pick C1-Like 1 (NPC1L1) [5], Margier et al. [11] demonstrated that D3 is also transported by the cholesterol efflux protein, ATP binding cassette subfamily A1 (ABCA1). From her results, she posited that D3 could be added to HDL particles in or around the enterocyte. This hypothesis is further substantiated by work conducted nearly 30 y prior by Haddad, which found 50%–60% of an oral pharmacologic dose of D2 appears in the portion of plasma containing HDL and protein (*d* >1.3 g/mL) [8]. This proposed pathway aligns with HDL's well-established function in reverse cholesterol transport, where it efficiently escorts excess cholesterol from peripheral tissues back to the liver for processing.

The small intestine is also recognized as a significant site of HDL synthesis, producing nascent HDL particles that are secreted into the lymphatic system [[12], [13], [14]]. Studies indicate that HDL particles originating in the intestine are smaller and possess different ratios of lipids and proteins compared with larger liver-derived HDL, potentially increasing their likelihood to carry VITD [15,16]. Although DBP is predominantly synthesized in the liver and a portion of systemic DBP is known to associate with lipoproteins in plasma [17], the nascent intestinal HDL particles are initially free of DBP [16]. Taken together, *1*) VITD's structural similarity to free cholesterol, *2*) VITD interaction with known enterocyte cholesterol transporters, and *3*) HDL's reported association with DBP, the role of HDL in VITD transport from the enterocyte warrants rigorous investigation. This is especially plausible in the intestine, as opposed to the liver, given the liver's immediate role in hydroxylating VITD to the longer-lived 25OHD metabolite.

## Potential HDL-Mediated VITD Delivery from Intestine to Liver

The liver is central to both cholesterol and VITD metabolism, serving as the primary site for the initial hydroxylation of VITD at the 25th carbon by CYP2R1, producing 25OHD [18,19]. Efficient delivery of VITD to the liver is of utmost importance for maintaining VitD status [19]. A key player in hepatic cholesterol uptake is SR-B1 transport protein, which facilitates the selective internalization of cholesterol from HDL particles [20]. Given that SR-B1 can also take up D3 from micelles in the gut [5], it is highly plausible that this same protein could mediate the liver's uptake of VITD if transported via HDL. The currently recognized primary carrier of VITD is DBP, which is made in the liver [4]. DBP exhibits a relatively low affinity for VITD but a high affinity for its hydroxylated metabolite, 25OHD, suggesting it is unlikely to be a major solo player in this initial transport from the intestine. Instead, DBP's primary role in transport likely becomes prominent after VITD’s conversion to 25OHD in the liver [4,21]. Thus, although DBP can circulate independently or associate with lipoproteins, its direct involvement in carrying the initial dietary VITD load to the liver could be predicted to be less significant compared with its role in the subsequent systemic distribution of 25OHD [4,15,21]. The interplay among the points above reveals the potentially overlooked role for HDL to facilitate initial delivery of VITD to the liver, which is supported by strong associations between VitD status and lifestyle factors and medications known to affect HDLc.

## Conditions Affecting HDL Cholesterol and Their Association with VitD Status

This proposed mechanism, where HDL serves as a VITD carrier from the intestine to the liver, offers a plausible explanation for observed correlations between VitD status and factors known to affect HDLc. Specifically, changes in circulating HDLc may alter the "carrying capacity" for VITD by affecting its transport to the liver for subsequent hydroxylation to 25OHD.

Several conditions that elevate plasma HDLc are concurrently associated with improved vitamin D status:

### Fibrate use

These PPAR-α activators, known for increasing HDLc, have been linked to improved VitD status. Makariou et al. [22] observed a 64% improvement in VitD status alongside increased HDLc in a statin-fibrate combination group (*P* < 0.001 compared with baseline, Table 1) [[22], [23], [24], [25], [26], [27], [28]], whereas Krysiak et al. [23] demonstrated that fibrate treatment led to an 8% increase in serum 25OHD concentrations in individuals with higher baseline VitD status (*P* < 0.05, Table 1). Both studies confirmed these improvements occurred without changes in VITD intake or sun exposure [22,23].

**TABLE 1:** Summary of drug studies showing parallel impacts on HDL and vitamin D status.

Author (y)	Participants	Methods/treatments	Results^1^^,^^2^	Comments
Fibrates				
Makariou et al. (2012) [22]	Adults (*n =* 60) Baseline unknown VitD status	Open label, baseline vs. medication (40 mg atorvastatin vs. 10 mg atorvastatin+200 mg fenofibrate) 3-mo treatment	HDL baseline 1.37; treatment 1.47 ↑ 7.7% *P* value = 0.02 vs. baseline VitD baseline 35.25; treatment status 46.0 ↑ 64% *P* value 0.001 vs. baseline	HDL and VitD status improve with fibrate treatment
Krysiak et al. (2021) [23]	Adult women (*n =* 61) Known VitD status - Deficient - Insufficient + Supplementation - Insufficient	Open label baseline vs. medication (200 mg fenofibrate) 6-mo treatment	VitD-deficient group: - HDL baseline 1.06; treatment 1.19 - 12% *P* value < 0.05 vs. baseline - VitD baseline 63; treatment 61 - ↓ Tre*P* value NS^3^ VitD insufficient+supplement group: - HDL baseline 1.06; 1.37 - ↑ 29% *P* value < 0.05 vs. baseline and vs. deficient group - VitD baseline 98; 107 - ↑ 9% *P* value < 0.05 vs. baseline VitD insufficient group: - HDL baseline 1.03; Treatment 1.31 - ↑ 27% *P* value < 0.05 vs. baseline and vs. deficient group - VitD baseline 100; 108 - ↑ 8% *P* value < 0.05 vs. baseline	- HDL status improved for all fibrate-treated groups, but more robustly with higher VitD status - VitD status improved with fibrate treatment without change in intake or supplementation
Ezetimibe				
Liberopoulos et al. (2013) [27]	Adult men and women (*n =* 50) Unknown VitD status	Open label, baseline vs. medication (40-mg simvistatin, vs. 10-mg simvastatin+ 10 mg ezetimibe) 12-mo treatment	Simvastatin+ezetimibe group - HDL baseline 1.53; treatment 1.55 - ↑ 1.6% NS^3^ - VitD baseline 17; treatment 23.25 - ↑ 37% *P* value < 0.000 vs. baseline Simvastatin group - HDL baseline 1.60; treatment 1.66 - ↑ 1.6% NS^3^ - VitD baseline 16.75; treatment 30 - ↑ 79% *P* value = 0.008 vs. baseline	HDL status did not improve with ezetimibe treatment VitD status decreased with ezetimibe treatment
Oral contraceptives (OC)				
Silva-Bermudez (2020) [28]	Premenopausal women	Meta-analysis of 82 studies including OC^4^ use for > 3 mo with lipid panel data	OC treatment mean ↑ 6.51 mg/dL HDL (CI: 3.12, 9.89), *P* value = 0.0002 vs. baseline	HDL status improves with OC treatment
Özcan et al. (2024) [24]	Women	Narrative review OC use 21 studies of HDL concentration, 5 studies VitD status, and 4 DBP^5^ studies	HDL: - 13 studies ↑ 8%–15% - 12 studies no change - 6 studies ↓ 8%–20% VitD Status - 4 studies ↑ 20%–41% - 1 study no change DBP - 2 studies ↑ 20%–25% - 1 study no change - 1 study ↓ 19%	HDL, VitD status, DBP improve with OC use
Callegari et al. (2017) [25]	Adolescent women (*n =* 407) 16–25 y	Cross-sectional observational study	VitD status: - No OC use: mean 60 nmol/L - OC use: mean 79 nmol/L - ↑ 32% *P* value < 0.001 vs. no use	VitD status improves with OC use
Stanczyk et al. (2021) [26]	Women (*n =* 27) 20–39 y	Retrospective pooled cross-sectional study OC treatment for 21 d	VitD status: - Baseline 42.25 nmol/L; treatment 43 nmol/L - ↑1% *P* value 0.65 vs. baseline - Baseline DBP 260 μg/mL	VitD status did not improve with 21-d OC treatment

Abbreviations: CI, confidence interval; DBP, vitamin D-binding protein; VitD, vitamin D.

1 HDLc units expressed as mmol/L; men > 1.0 mmol/L, women > 1.2 mmol/L normal.

2 VitD status expressed as nmol/L; deficient < 75 nmol/L; insufficient 75–125 nmol/L; sufficient > 125 nmol/L.

3 Nonsignificant.

4 Oral contraceptive.

5 Vitamin D-binding protein units expressed as μg/mL; reference value 200–350 μg/mL.

### Oral contraceptive use

Oral contraceptive (OC) use consistently correlates with elevated 25OHD concentrations and increased HDLc (Table 1). Reviews and meta-analyses affirm that OC use is associated with improved VitD status [24,29], with multiple studies showing concomitant increases in HDLc [25,26]. Although some research primarily attributes increased 25OHD to changes in DBP [26], these findings corroborate a broader connection between OC, HDLc, and VitD status that warrants further investigation.

### Exercise

Aerobic and endurance exercise are well-established enhancers of HDLc, positively influencing its particle characteristics and functionality [30,31]. What is less appreciated is that when VitD status is measured in these exercise studies, it is usually improved along with HDLc in both human and animal interventions [32]. Population-level studies reinforce this, showing positive associations between higher exercise levels and increased 25OHD concentrations [23,33,34]. Individual studies provide further nuance; Hansen et al. [35] found that within a VITD supplementation group, those with the greatest increase in 25OHD also performed more moderate-to-vigorous endurance exercise. Even a single bout of exercise can transiently elevate 25OHD, with sustained elevation observed for 24 h (Table 2) [25,31,33,35,[36], [37], [38], [39], [40], [41], [42], [43], [44]]. Although exercise-induced release of 25OHD from muscle or adipose tissue is one proposed mechanism for short-term effects [19,37], the sustained elevation shown in studies as reported by Malandish et al. [38], where long-term endurance training improved vitamin D status even after detraining, suggests a more complex, potentially transport-related mechanism.

**TABLE 2:** Summary of lifestyle studies showing parallel impacts on HDL and vitamin D status.

Author (y)	Participants	Methods/treatments	Results^1^^,^^2^	Comments
Smoking				
He et al. (2013) [39]		Narrative review	HDL active compared with nonsmokers	HDL concentration decreased with cigarette smoking
Yang et al. (2021) [40]	Adult men and women without diseases affecting VitD absorption or metabolism	Meta-analysis of 24 studies including smoking and vitamin D status endpoints	VitD status - Deficient RR 1.1 (CI: 1.03, 1.19) *P* value < 0.001 vs. nonsmokers - ↓Status (SMD −0.24, 95% CI: −0.31, −0.17), *P* < 0.001 smokers vs. nonsmokers	VitD status lower and more likely deficient with cigarette smoking
Exercise (EX)				
Franczyk et al. (2023) [31]		Narrative review	HDL ↑ 4.6% with prolonged low-to-moderate EX^3^	HDL increased with habitual endurance EX

Callegari et al. (2017) [25]	Women (*n =* 407) 16–25 y	Cross-sectional observational study	VitD status - Minimal-to-low EX 62 nmol/L - Moderate-to-high EX 70 nmol/L - ↑ 13% *P* value = 0.009	VitD status improves with habitual EX
Hibler et al. (2016) [33]	Adult men and women (*n =* 876) 40–80 y with history of treated colorectal adenoma	Retrospective pooled cross-sectional study with VitD and EX data	Odds VitD status greater deficient highest EX (3.45 95% CI: 2.22, 5.67) *P* value = 0.001 vs. lowest EX	VitD status improves with habitual EX
Hansen et al. (2021) [35]	Adult Institutionalized men (*n =* 67)	Randomized controlled VitD supplement (1600 IU) treatment with parallel EX group design	VitD status: - Baseline treatment 64.3 nmol/L; control 57.7 nmol/L - Postsupplement treatment 75.4 nmol/L; control 46.1 nmol/L - VitD treatment ↑ 17% *P* value < 0.001 vs. control - Postsupplement high treatment responders 4.6 EX scale; low treatment responders 2.5 EX scale *P* value < 0.001	VitD status improves more with supplementation and habitual EX
Dzik et al. (2022) [36]	Adolescent men (*n =* 26) 10–14 y	Randomized single bout EX trial	VitD status Baseline 72 nmol/L 15-min post EX 74.9 nmol/L ↑ (4%) *P* value = 0.016 vs. baseline 1-h post EX 75 nmol/L ↑ 4% *P* value = 0.011	VitD status improves with single bout EX
Sun et al. (2017) [37]	Adult men and women (*n =* 20) 18–22 y	Repeated measure single bout EX trial	VitD status: - Baseline 69 nmol/L; 24-h posttreatment 74.8 - ↑ 8.5% *P* value < 0.05 vs. baseline	VitD status improves with single bout EX
Malandish et al. (2020) [38]	Postmenopausal women (*n =* 26) 50–70 y	Randomized EX trial postmenopausal women 12 wk moderate-intensity training with 5 mo detraining	VitD status: - Baseline control 19.75; treatment 11.23 nmol/L - Training control 18.2; treatment 28.73 - ↑ 254% *P* value < 0.05 vs. baseline - ↑ 56% *P* value < 0.05 vs. control - Detraining control 16.5; treatment 31.3 - ↑ 282% *P* value < 0.05 vs. baseline - ↑ 11% NS^4^ vs. training	VitD status improves with habitual EX and remains improved with detraining
Song et al. (2020) [41]	Adolescent men and women (*n =* 3183) 12–18 y	KHANES cross section	- Men - Normal BMI+low HDL 81% population VitD deficient, ↑ *P* value 0.049 vs. normal HDL - > 25 BMI+Low HDL 83% population VitD deficient, ↑ *P* value 0.003 vs. normal HDL - VitD deficient+normal BMI ↓ 2 h EX *P* value 0.001 vs. VitD sufficient, *P* value = 0.011 vs. VitD sufficient - VitD deficient+>25 BMI ↓2 h EX *P* value 0.005 vs. VitD sufficient - Women - Normal BMI+Low HDL 67% population VitD deficient, ↓ *P* value 0.001 - > 25 BMI+Low HDL 73% population, *P* value 0.003 - VitD deficient+normal BMI i1.7 h EX *P* value NS vs. VitD sufficient - VitD deficient+>25 BMI e1.1 h EX *P* value NS vs. VitD sufficient	- Low HDL associated with VitD deficiency - VitD sufficiency associated with greater physical activity in men
Tsuprykov et al. (2021) [42]	Pregnant women (*n =* 427) > 18 y	Repeated measures cross section	HDL - 1st trimester 1.5 - 2nd trimester 1.68 ↑ 9.6% *P* value < 0.01 vs. 1st trimester - 3rd trimester 1.75 ↑ 14% *P* value < 0.001 vs. 1st trimester VitD - 1st trimester status 47 - 2nd trimester status 50.7 ↑ 7% NS vs. 1st trimester - 3rd trimester status 51.38 ↑ 9% NS vs. 1st trimester 0.228 Spearman’s rho correlating HDL and status, *P* value < 0.001	HDL concentration strongly correlated with VitD status in pregnancy
Tavakoli et al. (2016) [43]	Adolescent men and women (*n =* 40) 10–14 y	Randomized prospective single-blind VitD supplement (1000 IU)	HDL - Control baseline 1.17; 1-mo 1.12 - Treatment baseline 1.18; 1-mo 1.28 - ↑ 9% *P* value = 0.007 vs. baseline treatment and *P* value = 0.006 vs. control VitD status - Control baseline 24.30; 1-mo 23.15 - Treatment baseline 18.88; 1-mo 28.5 ↑ 52% *P* value < 0.001 vs. baseline treatment and control	HDL and VitD status improve with VitD supplementation
Sabico et al. (2023) [44]	Adult men and women	Prospective single-arm interventional study of 50,000 IU weekly (2 mo), biweekly (2 mo), then 1000 IU daily (2 mo).	HDL - Baseline 1.01; posttreatment 1.1 - ↑ 10% *P* value 0.001 vs. baseline VitD - Baseline status 32.5 nmol/L; posttreatment 63.3 nmol/L (deficient) - ↑ 94% *P* value < 0.001 vs. baseline	HDL and VitD improve with VitD treatment

Abbreviations: CI, confidence interval; DBP, vitamin D-binding protein; KHANES, Korean Health and Nutrition Examination Survey; RR, relative risk; SMD, standardized mean difference; VitD, vitamin D.

1 HDLc units expressed as mmol/L; men > 1.0 mmol/L, women > 1.2 mmol/L normal.

2 VitD status expressed as nmol/L; deficient < 75 nmol/L; insufficient 75–125 nmol/L; sufficient > 125 nmol/L.

3 Exercise.

4 Nonsignificant.

These correlations collectively support the hypothesis that factors increasing HDLc may enhance VITD's transport and subsequent conversion to 25OHD. Conversely, factors known to decrease HDLc or directly inhibit D3 absorption may consequently limit the delivery of VITD to the liver and its subsequent conversion to 25OHD.

### Smoking

Cigarette smoking is a long-established negative modulator of HDLc and its function, with particularly pronounced effects in women and a dose-dependent relationship in heavy smokers [39]. This aligns with findings from a meta-analysis by Yang et al. [40], who reported that smokers are consistently more likely to exhibit a deficient VitD status and have lower 25OHD concentrations compared with nonsmokers, irrespective of supplementation.

### Ezetimibe use

This pharmaceutical directly inhibits the NPC1L1 cholesterol transporter, thereby limiting dietary cholesterol absorption in the enterocyte. Importantly, its impact extends to D3: Reboul et al.s' [5] pioneering 2011 study demonstrated that ezetimibe significantly lowered D3 uptake by 20% in CaCo-2 cells and by 65% in mouse intestine explants. Further supporting this, Kiourtzidis et al. [45] reported markedly reduced blood D3 concentrations in ezetimibe-treated mice compared with controls, with similar reductions observed in whole-body homogenates (excluding VitD-rich tissues). Although these animal models showed a pronounced effect, human studies on ezetimibe's impact on VitD status have yielded less consistent results. Liberopoulos et al. [27] observed that although both statin and statin-ezetimibe combination therapies improved VitD status in patients with elevated LDL cholesterol, the addition of ezetimibe attenuated the increase in 25OHD concentrations (Table 1). Subsequent human studies have reported mixed effects [29], suggesting that inconsistencies in ezetimibe's impact in humans on VitD status could potentially stem from its varied effects on HDL metabolism and cholecalciferol's incorporation into this transport pathway.

## Population-Based Studies Investigating HDLc and VitD Status

Only a few published studies *directly* investigate the intricate interplay between HDL and VitD metabolism, all of which are population-level: Song et al. [41] found a significant link between deficient VitD status and lower HDLc in Korean adolescents, and Tsuprykov et al. [42] reported a positive correlation between HDLc and total 25OHD concentrations in pregnant women (Table 1). Furthermore, VITD supplementation studies show concurrent increases in both 25OHD and HDLc in Iranian children [43] and Saudi adults [44], even without reported changes in medications or lifestyle.

## Unanswered Questions and Future Directions

Although the examples above provide compelling support for a role for HDL in VITD transport and the impact on VitD status (serum concentration of 25OHD), several limitations and gaps remain. A significant limitation of the existing evidence is that it is largely correlative in nature. The scarcity in human intervention data is attributable to several factors, further compounded by the fact that most VitD status assessments rely on measuring 25OHD, rather than the parent VITD. The dynamic nature of HDL, the relatively short half-life of VITD compared with its hydroxylated metabolites, and a multitude of confounding factors prevalent in human VitD status studies all contribute to this challenge. These confounders include person-intrinsic factors such as metabolic syndrome, BMI, age, and skin melanin content; lifestyle and behavioral habits such as dietary choices, supplement use, sunscreen application, and outdoor time; geographic and environmental influences encompassing latitude, seasonality, and air pollution; and broader socioeconomic factors affecting access to and acceptance of fortified foods, outdoor activity opportunities, and safety concerns. Each of these elements can independently or synergistically influence VitD status, and their complex interplay makes it exceptionally challenging to isolate the specific effects of HDL-mediated VITD transport in observational or interventional human studies, particularly when most studies measure 25OHD as the primary outcome]

To definitively elucidate the proposed mechanisms, future investigations should address several questions regarding cholecalciferol transport and metabolism:

1. *Post-absorption distribution of VITD*: Following a food dose challenge and subsequent intestinal absorption, how is VITD precisely distributed among plasma lipoprotein fractions (e.g., TRL, HDL) and/or protein fractions?
2. *HDL's direct influence on VITD*: How does HDLc directly affect VITD trafficking and its delivery to the liver?
3. *Impact of VitD status and dose*: How does an individual's baseline VitD status and the administered dose of VITD affect its trafficking patterns within these lipoprotein and protein fractions?
4. *HDL’s role in the trafficking UVB-skin synthesized D3*: Given what we know about the role of HDL in the disposal of cholesterol from peripheral tissues and the low binding affinity of DBP for D3, could HDL play a role in the trafficking of UVB-synthesized D3 in the skin (i.e. a recapitulation of the proposed intestinal transport)?

In conclusion, the widespread functions of VITD in human health underscore the need for a complete understanding of VITD's metabolism, beginning with its journey from the gut or other tissues to the liver. Although classical models emphasize chylomicron-mediated transport, compelling evidence from structural similarities with cholesterol, direct interaction with cholesterol transporters, and the reported presence of VITD in the HDL fraction challenge this sole pathway. We propose that HDL serves as an interorgan carrier for VITD, facilitating its movement from the intestine via known cholesterol transporters and its subsequent delivery to the liver for 25-hydroxylation, potentially utilizing hepatic cholesterol transporters such as SR-B1. This mechanism also provides a plausible explanation for the observed correlations between VitD status and various factors that modulate HDLc.

Although human intervention studies are needed to establish causation, the strong biochemical parallels and emerging correlative data present a robust argument for re-evaluating VITD's absorption and transport. A deeper understanding of HDL's role in VITD delivery holds significant implications for optimizing VitD status.

## Author contributions

Both authors authored the paper and approved the final manuscript.

## Declaration of generative AI and AI-assisted technologies in the writing process

During the preparation of this JDB used Gemini AI/Google Workspace for the first-round editing. After using this tool/service, the authors reviewed and edited the content substantially and take full responsibility for the content of the publication.

## Funding

JDB supported by the American Dissertation Fellowship from the American Association of University Women.

## Conflict of interest

The authors report no conflicts of interest.
